# Differential analysis of culturable and unculturable subgingival target microorganisms according to the stages of periodontitis

**DOI:** 10.1007/s00784-023-04907-5

**Published:** 2023-02-18

**Authors:** Gloria Inés Lafaurie, Diana Marcela Castillo, Margarita Iniesta, Mariano Sanz, Luz Amparo Gómez, Yormaris Castillo, Roquelina Pianeta, Nathaly Andrea Delgadillo, Yineth Neuta, David Diaz-Báez, David Herrera

**Affiliations:** 1 Unit of Basic Oral Investigation (UIBO), School of Dentistry, Universidad El Bosque, Ak. 9 #13, 1a-20 Bogotá, Colombia; 2 ETEP (Etiology and Therapy of Periodontal and Peri-Implant Diseases) Research Group, School of Dentistry, University Complutense of Madrid (UCM), Madrid, Spain; 3 School of Dentistry, Corporación Universitaria Rafael Núñez, Cartagena, Colombia

**Keywords:** Periodontitis, Bacteria, Diagnosis, Biofilm, Quantitative PCR

## Abstract

**Objectives:**

Culturable and unculturable microorganisms have been associated with periodontitis. Their differential proportions and composition have not been evaluated by their severity and complexity defined by stages in the 2018 AAP-EEP classification.

**Methods:**

One hundred eighty subgingival biofilm samples were collected in Spain and Colombia from subjects categorized as health/gingivitis: periodontitis stages I/II periodontitis stages III/IV. Target culturable microorganisms (*Porphyromonas gingivalis*, *Aggregatibacter actinomycetemcomitans, Tannerella forsythia*, *Treponema denticola*, and *Eubacterium nodatum)* and target unculturable microorganisms (*Filifactor alocis*, *Eubacterium saphenum*, *Eubacterium brachy*, *Desulfobulbus oralis)* were evaluated by quantitative PCR analysis. In addition, their differences and association with periodontal status were analyzed by ANCOVA and logistic regression models once adjusted to age, current smoking, and country.

**Results:**

*P. gingivalis* was significantly associated with periodontitis stages I/II, OR 2.44 (CI 95% 1.08–5.47) and stages III/V, OR 6.43 (CI 95% 2.43–16.9). *T forsythia*, OR 7.53 (CI 95% 2.07–27.4); *D. oralis*, OR 5.99 (CI 95% 2.71–13.23); *F. alocis*, OR 10.9 (CI 95% 4.56–23.2); *E. brachy*, 3.57 (CI 95% 1.40–9.11); and *E. saphenum*, 4.85 (CI 95% 1.99–11.7) were significantly associated only with stages III/IV periodontitis. *P. gingivalis* evidenced significant differences with the increase in the severity of the periodontal lesion: 2.97 colony forming unit (CFU)/μL (CI 95% 2.32–3.54) health/gingivitis, and 4.66 CFU/μL (CI 95% 4.03–5.30) and 5.90 CFU/μL (CI 95% 5.20–6.48) in stages I/II and III/IV respectively (*p* < 0.0001). Unculturable microorganisms only evidenced differences in concentration in stages III/IV compared with health-gingivitis (*p* ≤ 0.001).

**Conclusion:**

Culturable and unculturable are strongly associated with stages III/IV periodontitis. Classic culturable microorganisms are more sensitive to differentiate between stages of periodontitis in the quantitative analysis.

**Clinical relevance:**

Future interventional studies of periodontal disease should include *Filifactor alocis*, *Eubacterium saphenum*, *Eubacterium brachy*, and *Desulfobulbus oralis* as possible markers of therapy response and as indicators of progressive disease.

**Supplementary Information:**

The online version contains supplementary material available at 10.1007/s00784-023-04907-5.

## Introduction

The current classification of periodontal diseases (2018) is based on clinical and radiographical criteria using a multidimensional staging and grading system where severity, complexity, and progression of the disease, as well as the presence of proven risk factors, are the main differentiating criteria [1, 2]. Although periodontitis is currently defined as a multifactorial chronic inflammatory disease associated with dysbiosis of the subgingival biofilm, there is insufficient evidence to establish a characteristic microbiota associated with it or its different phenotypes [3]. When analyzing the differences in the predominant subgingival microbiota in periodontitis, the dysbiotic changes would be related to the transition of some bacteria from being associated with health to proinflammatory commensals and the overgrowth of specific cultivable bacteria such as *Porphyromonas gingivalis*, *Aggregatibacter actinomycetemcomitans*, *Tannerella forsythia*, and *Treponema denticola*. These specific cultivable bacteria are characterized by a close genetic homology and the ability to activate the innate immune response, thus triggering unresolved and destructive chronic inflammatory processes of periodontal supporting tissues [4, 5]. However, the differential presence of these putative periodontal pathogens has not been associated with a specific periodontitis phenotype [6]. New metagenomic technologies have reported the presence of high proportions of unculturable or difficult-to-cultivate bacteria in the subgingival biofilm, such as the phyla *Firmicutes*, *Bacteroidetes*, *Chloroflexi Tenericutes*, *Fusobacteria*, *Proteobacteria*, *Actinobacteria,* Spirochaetes, and Synergistetes [7–9]. Similar to culturable bacteria, some microorganisms of these phyla have been especially associated with periodontitis, such as *Filifactor alocis*, *Eubacterium saphenum*, *Eubacterium brachy*, *Peptostreptococcus*, and *Desulfobulbus oralis* [10–12]. However, information is lacking on whether these unculturable bacteria may be present differently in periodontitis phenotypes or whether there are variations according to geographic location. It was, therefore, the aim of this observational clinical and microbiological study to evaluate the differential presence of culturable microorganisms such as *P. gingivalis*, *T. forsythia*, *T. denticola*, *A. actinomycetemcomitans*, *F. nucleatum*, and *E. nodatum*, versus unculturable or difficult to cultivate microorganisms such as *F. alocis*, *E. saphenum*, *E. brachy*, and *D. oralis* in subjects from Spain and Colombia, categorized according to their periodontal status using the new classification of periodontal diseases (2018) [1–3].

## Material and methods

### Type of study

This cross-sectional clinical and microbiological observational study was reported following the STROBE criteria after being approved by the respective Clinical Ethics Committees (approval 012-2018 in Colombia and 18/127-E in Spain) following the international ethical guidelines of the Declaration of Helsinki regarding human experimentation.

### Sample size

**Fig. 1:**
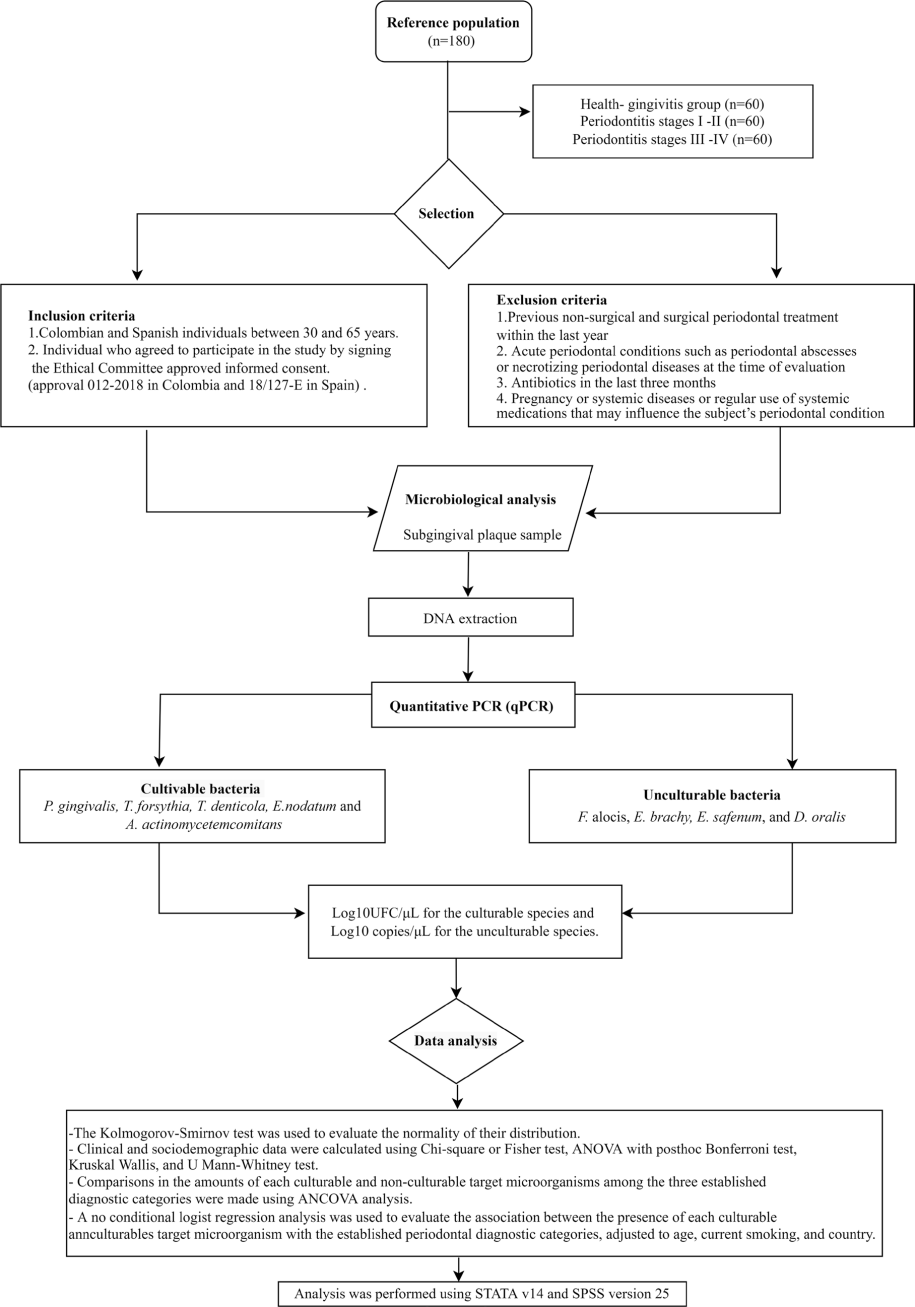
Flow chart of study design

The sample size was calculated based on an exploratory study with 50% of de samples of this study. A total sample of 170 subjects was estimated using Fleiss with continuity correction formula based on an odds ratio (OR) ≥ 3 for a minor proportion of 25% of microorganisms in the periodontal health/gingivitis group and 50% in the periodontitis groups, with an inter-group difference of 25%, for a 95% two-tailed confidence level and a power of 85%. Finally, 180 subgingival samples were harvested from subjects that were categorized according to their periodontal status, defined by the criteria of the classification of periodontal diseases (2018) [1] in three groups: health-gingivitis group (*n* = 60), periodontitis, stages I–II (*n* = 60), and periodontitis, stages III–IV (*n* = 60) (Fig. 1). This sample was equally represented by subjects between 30 and 65 years from Spain and Colombia matched by age (difference of no more than 5 years), periodontal condition, and country, who agreed to participate in the study by signing the Ethical Committee-approved informed consent. These subjects filled out a medical questionnaire including smoking habits and had a full mouth periodontal examination including recording of pocket depth (PD), clinical attachment loss (CAL) in six sites per tooth, as well as evaluation of radiographic bone loss (RBL) from periapical radiographs. Two calibrated examiners demonstrating interclass correlation coefficients (ICC) of 90.2% agreement for PD and 89% for CAL in Colombia, and 86.3% and 84.7% in Spain, respectively, established their periodontal diagnosis according to the 2018 classification. Once diagnosed, subjects were categorized into three groups: (1) periodontal health or gingivitis subjects, defined by no clinical attachment loss (CAL) or radiographic bone loss (RBL), and pocket depth (PD) ≤ 3 mm, or, periodontal health or gingivitis subjects, defined by no CAL or RBL, and PD ≤ 3 mm; (2) stages I or II periodontitis with evidence of interdental CAL of 1–2 mm (stage I) or 3–4 mm (stage II) and RBL that affects only the coronal third of the root; and (3) stages III or IV periodontitis with evidence of interdental CAL > 5 mm and RBL extending to the middle or apical third of the root.

Subjects were excluded in having the following criteria: (1) previous non-surgical and surgical periodontal treatment within the last year; (2) acute periodontal conditions such as periodontal abscesses or necrotizing periodontal diseases at the time of evaluation; (3) antibiotics in the last 3 months; and (4) pregnancy or systemic diseases or regular use of systemic medications that may influence the subjects’ periodontal condition.

In periodontal health/gingivitis, samples were taken from the mesiobuccal sites of the first molars and, when absent, from the adjacent second molars. In periodontitis, subgingival samples were taken from the most accessible site with the deepest PD and BoP in each quadrant. Samples were taken from four selected sites by placing two consecutively sterile paper points (Maillefer, Ballaigues, Switzerland) per site, which were left at the bottom of the sulcus/pocket for 10 s. All paper points were transferred into a screw-capped vial containing 1.5 mL of reduced transport fluid (RTF) and sent to the microbiological laboratories of each center within 24 h. At the laboratories, samples were initially plated on culture media [13], and samples in RTF medium were frozen until further processing. Frozen samples from Spain were sent to the laboratory in Colombia.

### DNA extraction

After thorough vortexing, 200 μL of the microbial suspension was washed three times in molecular grade distilled water, resuspended in 100 μL of distilled water, boiled for 10 min, and placed on ice. After centrifugation to remove cell debris, the supernatant was used for PCR analysis.

### Quantitative PCR for the detection of cultivable bacteria

Quantitative PCR (qPCR) to detect *P. gingivalis*, *T. forsythia*, *T. denticola*, *E. nodatum*, *F*. *nucleatum*, and *A. actinomycetemcomitans* was performed using the following primers and TaqMan probes. For *P. gingivalis*, the reaction mixture contained 3 mM MgCl_2_, 1× buffer )GoTaq Polymerase®) (Promega. Wisconsin, USA), 0.1 mM dNTPs, 0.9 μM of the specific probe, 1 μM of primers, and GoTaq Polymerase® DNA G2 (Promega. Wisconsin, USA) 0.06 UI/μL, according to Boutaga et al. 2003 [14]. For *A. actinomycetemcomitans*, the reaction mixture contained 3 mM MgCl_2_, 1X buffer (GoTaq Polymerase®) (Promega. Wisconsin, USA), 0.2 mM dNTPs, 0.5 μM probe specific for *A. actinomycetemcomitans*, first sense 0.3 μM, first antisense 0.9 μM, and DNA GoTaq Polimerase® G2 (Promega. Wisconsin, USA) 0.06 UI/μL according to Boutaga et al. (2005) [15]. *T. forsythia* was identified using the protocol reported by Morillo et al. (2004) [16], and the concentrations adjusted for the reaction mixture constituted 3.5 mM MgCl_2_, 1× buffer (GoTaq Polymerase®) (Promega. Wisconsin, USA), dNTPs at a concentration of 0.2 mM, primers 2 μM, and 0.5 μM probe specific for *T. forsythia* and DNA GoTaq Polymerase® G2 (Promega. Wisconsin, USA) 0.06 UI/ μL. For the identification of *T. denticola*, we used the protocol reported by Yoshida et al. (2004) [17] with a reaction mixture containing 2.25 mM MgCl_2_, 1× buffer (GoTaq Polymerase®) (Promega. Wisconsin, USA), 1.5 mM dNTPs, 0.5 μM primers, and 0.5 μM specific probe for this bacterium and DNA GoTaq Polimerase® (Promega. Wisconsin, USA) 0.125 U. The identification of *E. nodatum* was previously standardized by our group [18]; the standardized conditions included amplification with 3 mM MgCl_2_, 1× buffer (GoTaq Polymerase®) (Promega. Wisconsin, USA), 0.1 mM dNTPs, 1 μM primers, 0.5 μM specific probe, GoTaq Polymerase® DNA G2 (Promega. Wisconsin-USA) 0.06 UI/ μL.

*F. nucleatum* primer sequences were reported by Cross et al. (2018) [19], and the TaqMan probe was designed by our group (data in the process of publication). The amplification conditions were standardized to final concentrations of 2.0 mM MgCl_2_, 1× buffer (GoTaq Polymerase®) (Promega. Wisconsin, USA), 0.1 mM dNTPs, 0.3 μM primers, 0.3 μM specific probe, GoTaq Polymerase® DNA G2 (Promega. Wisconsin, USA) 0.06 UI/ μL. For amplification, 1 uL of previously quantified DNA was used. All samples were amplified in a CFX 96 thermocycler (BioRad® California, USA) and subjected to an initial amplification cycle of 95 °C for 10 min, followed by 45 cycles at 95 °C for 15 s and 60°C for 1 min. Quantification was carried out from calibration curves made for each of the bacteria with DNA samples from reference strains: *P. gingivalis* (ATCC 33277), *A. actinomycetemcomitans* (ATCC 29522), *T. forsythia* (ATCC 43037), *T. denticola* (ATCC 35405), *E. nodatum* (ATCC 33099), and *F. nucleatum* (ATCC 25586) with a known number of bacteria CFU)/mL. Results were expressed in logarithm base 10 (Log10) transformed CFU/mL.

### qPCR for the detection of unculturable bacteria

TaqMan primers and probes were designed to target the 16S rRNA gene from *E. brachy* (GenBank accession number: MT45929), *E. saphenum* (GenBank accession number: NR_026031.1), and the probe by *D. oralis* (GenBank accession number: GU398182.1) by Beacon Designer™ 8 software (Premier Biosoft). The primers and probe for *F. alocis* used were those reported by Al-hebshi et al. (2015) [20] and the primers by *D. oralis* were also previously reported [19]. The specificity of the primers and hydrolysis probes were verified in silico by BLAST analysis (National Library of Medicine https://blast.ncbi.nlm.nih.gov/Blast.cgi).

All qPCR reactions were performed using a CFX96 Thermal cycler (Bio-Rad®. California, USA). Each reaction consisted of 2.5 mM MgCl_2_, 1× buffer (GoTaq Polymerase®) (Promega. Wisconsin, USA), 0.1 mM dNTPs, 0.04 μM primers, 0.03 μM specific probe, GoTaq Polymerase® DNA G2 (Promega. Wisconsin, USA) 0.06 UI/ μL. For amplification, 1 uL of previously quantified DNA was used.

The quantification curves were standardized for each target microorganism using plasmids and the plasmids containing the fragment of interest were amplified using *Escherichia coli* TOP10. The amplified products were cloned into the pGEM®T Easy vector (Promega. Wisconsin, USA), extracted, and quantified for producing the standard quantification curves. The extracted plasmid DNA was then quantified in ng/μL by using the Implen® NanoPhotometer (Cole-Parmer. Vernon Hills, USA) and then converted to copies/μL as described by Hoorzook et al. (2021) [21].

Plasmid DNA from each of the target bacteria was used as a positive control for the qPCR amplification and results were expressed in CFU/μL that were subsequently transformed into Logarithm base 10 (Log10).

### Data analysis

Quantitative data from the qPCR analysis were expressed in Log10 CFU/μL for the culturable species and Log10 copies/μL for the unculturable species. The Kolmogorov-Smirnov test was used to evaluate the normality of their distribution. Absolute and relative frequencies were used to estimate the categorical variables. Clinical and sociodemographic data were calculated based on periodontal groups and countries and compared using chi-square or Fisher’s test, ANOVA with post hoc Bonferroni test, Kruskal-Wallis, and Mann-Whitney *U* test. Comparisons in the amounts of each culturable and unculturable target microorganisms among the three established diagnostic categories were made using ANCOVA analysis. A no-conditional logistic regression analysis was used to evaluate the association between the presence of each culturable and unculturable target microorganism with the established periodontal diagnostic and for categories, adjusted to age, current smoking, and country. The adjusted and unadjusted models were verified by the goodness-of-fit test and compared using the likelihood-ratio chi-square test (G2) and the Bayesian information criterion (BIC) [22]. Analysis was performed using STATA v14 (StataCorp LLC. USA) and SPSS version 25 (SPSS Inc. Chicago, IL, USA).

## Results

### Clinic characterization

Table 1 depicts the descriptive clinical characteristics of the selected sample population, which was matched for periodontal stage and age. However, the Spanish sample had a significantly higher frequency of smokers (*p* = 0.006), especially in the periodontitis stage III–IV category (*p* = 0.012). Although the three clinical categories (health/gingivitis, periodontitis stages I/II, and periodontitis stages III/IV) were pre-established, the subjects from Colombia demonstrated higher plaque levels in the health-gingivitis group (*p* = 0.0001) and higher BoP in the periodontitis stages I/II group (*p* = 0.003). Although subjects from both countries were matched for the three clinical categories, subjects in the periodontitis stage III–IV in Spain had more severe CAL (*p* = 0.002).

**Table 1:** Sociodemographic characteristics by country and periodontal status

	Periodontal status			Difference among condition	Difference between countries
	Periodontal health/gingivitis *n* = 60	Periodontitis stages I–II *n* = 60	Periodontitis stages III–IV *n* = 60	*p*-value	*p*-value
Country (*n*)					
Colombia	30	30	30	**¥**1.00	§1.00
Spain	30	30	30		
Gender					
Female/Male (*n* (%))					
Colombia	19 (40)/11 (26)	15 (36)/15 (31)	16 (38)/14 (29)	**¥**0.90	§0.88
Spain	20 (39)/10 (26)	14 (36)/16 (31)	15 (38)/15 (29)		
Current smoker					
Smokers (*n* (%))				**¥ 0.006**	**§0.006**
Colombia	2 (6.7)	2 (6.7)	5 (16.7)		
Spain	3 (10)	7 (23)	19 (43)		^a^0.64
No smokers (*n* (%))					^b^0.07
Colombia	28 (93.3)	28 (93.7)	25 (83.3)		^**c**^**0.024**
Spain	27 (90)	23 (77)	17 (57)		
Grades (*n* (%)					
Grade A					
Colombia	NA	11 (91.7)	1 (8.3)	**¥0.0001**	**§**0.57
Spain	NA	8 (100)	0 (0)		
Grade B					^b^0.13
Colombia	NA	13 (52)	12 (48)		^c^0.06
Spain	NA	20 (83.3)	4 (16.7)		
Grade C					
Colombia	NA	6 (26)	17 (74)		
Spain	NA	2 (7)	26 (93)		
**Age (mean (SD))**				**¥0.89**	§0.97
Colombia	45.4 (8.5)	45.1 (9)	46 (8.8)	^**a**^**0.98**	^a^0.84
Spain	45.4 (10.7)	45.5 (8)	45.8 (8.1)	^**b**^**0.96**	^b^0.96
				^**c**^**0.92**	^c^0.89
Plaque index (PII)				**¥0.0001**	**§0.002**
Median (IQR)					
Colombia	19 (10-33)	50 (24-99)	93 (66-100)	^**a**^**0.001**	^**a**^**0.0001**
Spain	54 (38-68)	69 (40-85)	85 (58-100)	^**b**^**0.0001**	^b^0.448
				^**c**^**0.007**	^c^0.737
Bleeding on probing (BoP)				**¥0.0001**	**§0.02**
Median (IQR)					
Colombia	11 (4-15)	59 (33-100)	100 (70-100)	^**a**^**0.0007**	^**a**^**0.595**
Spain	13 (4-27)	32 (17-45)	61 (38 -81)	^**b**^**0.0001**	^**b**^**0.003**
				^**c**^**0.0001**	^**c**^**0.014**
Pocket depth (PD) Mean (SD)				**¥0.000**	§0.27
Colombia	2.2 (0.2)	2.9 (0.6)	3.7 (0.6)	^**a**^**0.0001**	^**a**^0.391
Spain	2.2 (0.3)	2.9 (0.3)	4.0 (0.7)	^**b**^**0.0001**	^**b**^0.906
				^**c**^**0.0001**	^**c**^0.069
Clinical attachment loss Mean (SD)				**¥0.0001**	§0.42
Colombia	0.40 (0.3)	2.7 (1)	4.4 (0.9)	^**a**^**0.0001**	^a^0.257
Spain	0.33 (0.2)	3.0 (0.4)	4.8 (0.8)	^**b**^**0.0001**	^b^0.105
				^**c**^**0.0001**	^c^0.094

*n* number of patients, *SD* standard deviation, *IQR* interquartile range, *NS* no statistically significant differences (*p*>0.05)

¥=differences among groups including the entire population (stages of periodontitis and periodontal health/gingivitis group)

^a^Differences between periodontal health/gingivitis and stages I–II periodontitis

^b^Dfferences between periodontal health/gingivitis and stages III–IV periodontitis

^c^Differences between stages I–II and stages III–IV periodontitis

The column of differences between countries compares each periodontal category between countries §=differences among countries, a, b, c are differences among stages

Statistically, significant differences are marked in bold

### Prevalence of culturable and unculturable microorganisms by stages of periodontitis and countries

**Fig. 2:**
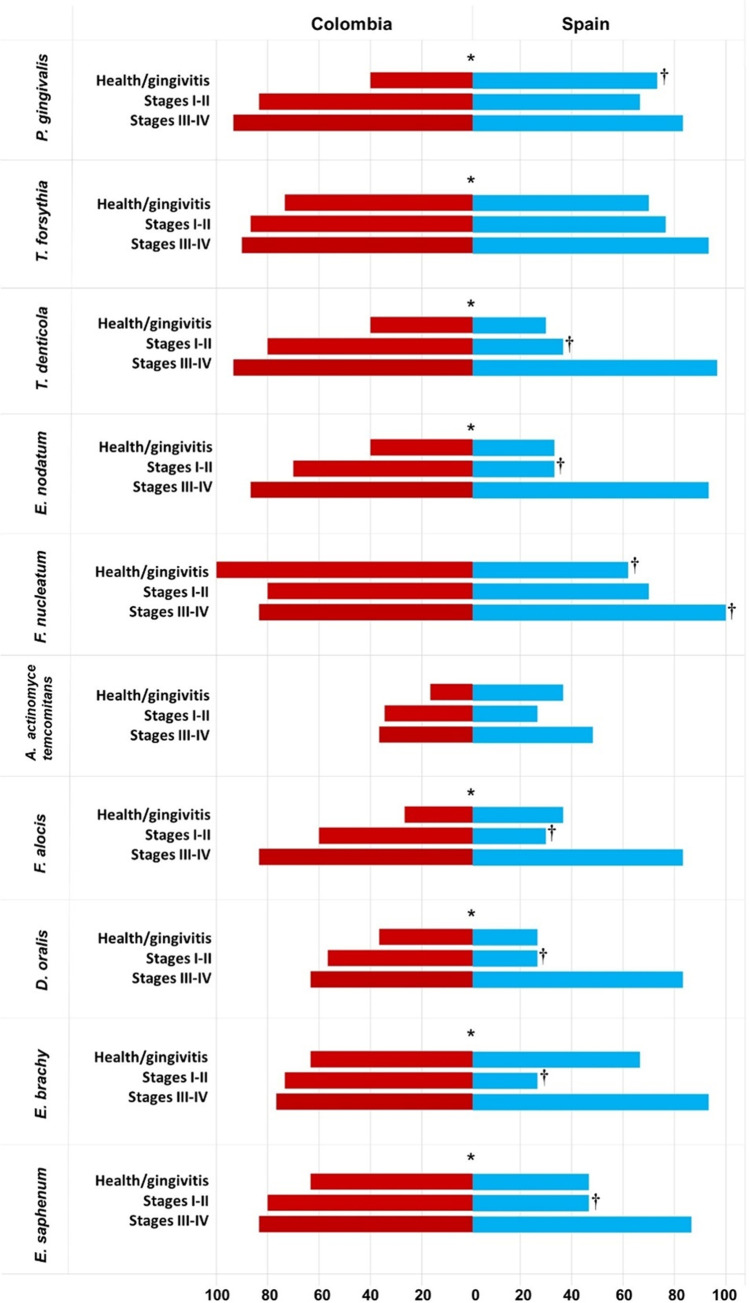
Qualitative analysis: frequency of detection of culturable and unculturable microorganisms, by stages and country. *Statistically significant differences among periodontal health status groups; ^†^Statistically significant differences among stages, by countries

Figure 2 depicts the prevalence of culturable and unculturable microorganisms. Target culturable bacteria such as *P. gingivalis*, *T. forsythia*, *T. denticola*, and *E. nodatum*, as well as unculturable microorganisms such as *F. alocis*, *E. saphenum*, *E. brachy*, and *D. oralis* were present at a significantly higher prevalence in periodontitis subjects in both stages I/II and stages III–IV when compared with the health-gingivitis group in both countries (*p* < 0.0001). *A. actinomycetemcomitans* and *F. nucleatum* had similar prevalences in periodontitis subjects irrespective of the staging and location (country) (*p* > 0.05). However, *P. gingivalis* (40% vs. 73%), *A. actinomycetemcomitans* (11% vs. 5%), and *F. nucleatum* (62% vs. 100%) had a significantly higher prevalence in Colombians subjects in the health-gingivitis group compared with the Spanish group. *E. nodatum* (33% vs*.* 70%), *T. denticola* (37% vs. 80%), and all unculturable microorganisms such as *F. alocis* (30% vs. 60%), *D. oralis* (27% vs. 57%), *E. brachy* (27% vs.73%), and *E. saphenum* (47% vs. 80%), were also in higher prevalence in Colombian periodontitis subjects with stages I/II (*p* < 0.05). *P. gingivalis* was higher in Colombian periodontitis subjects compared with Spanish individuals with stages III/IV (83% vs. 93%) (*p* < 0.05), other culturable or unculturable microorganisms did not show a higher prevalence in any of the countries in advanced stages of the disease.

### Multivariate analyses of the presence of culturable and unculturable microorganisms in periodontitis

Supplementary Table 1 (Table S1) depicts the multivariate analysis assessing the probability of the presence of culturable and unculturable microorganisms in periodontitis subjects. Culturable and unculturable microorganisms were associated with periodontitis presence adjusted to confounder variables. *P. gingivalis*, *T. denticola*, and *E. saphenum* also were associated with the country (more frequently for Colombian samples). Table 2 shows the analysis when it was established by stages compared with the health/gingivitis group as control, once adjusted by smoking habit, age, gender, and country. The presence of *P. gingivalis* was significantly associated with stages I/II, OR 2.44 (1.08–5.47) and periodontitis stages III–IV, OR 6.43 (2.43–16.9). *T. forsythia*, OR 7.53 (2.07–27.4); *D. oralis*, OR 5.99 (2.71–13.23); *F. alocis*, OR 10.9 (4.56–23.2); *E*. *brachy*, 3.57 (1.40–9.11); and *E. safenum*, 4.85 (1.99–11.7) were only significantly associated with stages III–IV periodontitis. Only *P. gingivalis* 3.22 (1.53–6.76) and *E. safenum* 2.18 (1.22–4.79) demonstrated a differential geographical association being both microorganisms were more frequent in Colombian subjects.

**Table 2:** Multivariate analysis by non-conditional logistic regression for the detection of culturable and unculturable microorganisms by periodontal health status and country

	Independent variables	Unadjusted OR (95% CI)	Adjusted OR (95% CI)	LR (*p-*value) BIC
*P. gingivalis*	Country			
	Spain	1.0 (Ref.)	1.0 (Ref.)	
	Colombia	3.22 (1.53–6.76)	3.84 (1.38–10.74)	
	Groups			
	Health/gingivitis	1.0 (Ref.)	1.0 (Ref.)	LR (chi^2^) = 0.62 Unadjusted BIC = 203.28 Adjusted BIC = 208.23 Difference = − 4.95
	Stages I–II	2.44 (1.08–5.47)	2.71 (1.09–7.72)	
	Stages III–IV	6.43 (2.43–16.9)	7.68 (2.28–25.8)	
*T. forsythia*	Groups			
	Health/gingivitis	1.0 (Ref.)	1.0 (Ref.)	LR (chi^2^) = 0.35 Unadjusted BIC = 172.91 Adjusted BIC = 181.20 Difference =−8.29
	Stages I–II	1.76 (0.74–2.86)	1.73 (0.72–4.14)	
	Stages III–IV	7.53 (2.07–27.4)	7.01 (1.88–26)	
*T. denticola*	Country			
	Spain	1.0 (Ref.)	1.0 (Ref.)	
	Colombia	2.55 (1.24–5.23)	2.33 (0.82–6.57)	
	Groups			
	Health/gingivitis	1.0 (Ref.)	1.0 (Ref.)	LR (chi^2^) = 0.35 Unadjusted BIC = 204.43 Adjusted BIC = 209.54 Difference =−8.29
	Stages I–II	2.53 (1.17–5.46)	2.36 (0.92–6.18)	
	Stages III–IV	21.6 (7.27–77.45)	19.5 (4.94–73.3)	
*E. nodatum*	Groups			
	Health/gingivitis	1.0 (Ref.)	1.0 (Ref.)	LR (chi^2^) = 0.35 Unadjusted BIC= 204.43 Adjusted BIC = 209.54 Difference =−5.11
	Stages I–II	2.01 (0.81–5)	2.01 (0.81–5)	
	Stages III–IV	15.6 (4.49–67.3)	17.4 (5.7–42.7)	
*D. oralis*	Groups			
	Health/gingivitis	1.0 (Ref.)	1.0 (Ref.)	LR (chi^2^) = 0.62 Unadjusted BIC = 241.59 Adjusted BIC = 252.88 Difference = -11.29
	Stages I–II	1.54 (0.74–4.18)	1.74 (0.68–4.4)	
	Stages III–IV	5.99 (2.71 -13.23)	7.61 (2.11–20.4)	
*F. alocis*	Groups			
	Health/gingivitis	1.0 (Ref.)	1.0 (Ref.)	LR (chi^2^) = 0.82 Unadjusted BIC = 231.33 Adjusted BIC = 246.01 Difference =−14.68
	Stages I–II	1.74 (0.83–2.74)	1.48 (0.83–2.74)	
	Stages III–IV	10.9 (4.56–23.2)	7.72 (2.00–28.9)	
*E. brachy*	Groups			
	Health/gingivitis	1.0 (Ref.)	1.0 (Ref.)	LR (chi^2^) = 0.31 Unadjusted BIC = 203.28 Adjusted BIC = 208.23 Difference =−4.95
	Stages I–II	0.53 (0.25–1.11)	0.56 (0.25–2.11)	
	Stages III–IV	3.57 (1.40–9.11)	5.38 (1.30–12.11)	
*E. saphenum*	Country			
	Spain	1.0 (Ref.)	1.0 (Ref.)	
	Colombia	2.18 (1.22–4.79)	2.42 (1.22–4.79)	
	Groups			
	Health/gingivitis	1.0 (Ref.)	1.0 (Ref.)	LR (chi^2^) = 0.30 Unadjusted BIC = 227.50 Adjusted BIC = 235.49 Difference =−7.99
	Stages I–II	1.43 (0.67–3.01)	1.36 (0.64–2.89)	
	Stages III–IV	4.85 (1.99–11.7)	4.18 (1.69–10.3)	

Model adjusted for country, age, and smoking

*OR* odds ratio, *95% CI* 95 % confidence interval, *LR* likelihood ratio test, *BIC* Bayesian information criterion

All models showed no differences in the likelihood ratio test. The unadjusted model should be reported for all models because they present a lower BIC^22^

### Quantitative analysis of culturable and unculturable microorganisms in different stages of periodontitis

In the multivariate analysis by ANCOVA (Table 3), all tested microorganisms demonstrated higher quantities when comparing periodontitis stages III/IV and the health-gingivitis group (*p* < 0.05). However, only culturable microorganisms such as *P. gingivalis*, *E. nodatum*, and *T. denticola* evidenced differences among health/gingivitis and stages I/II. Unculturable microorganisms except for *E. saphenum* could not differentiate between healthy/gingivitis and milder stages of periodontitis. *P. gingivalis*, *T. forsythia*, *T. denticola*, and *E. saphenum* were also in significantly higher concentrations when comparing periodontitis stage III/IV versus stage I/II (*p* < 0.0001). On the contrary, *E. nodatum*, *F. nucleatum*, *A. actinomycetemcomitans*, and unculturable microorganisms such as *F. alocis* and *E. brachy* did not differentiate between stages of periodontitis (Table 3).

**Table 3:** Multivariate analysis of the comparison of the levels of each bacterial species expressed as mean and 95% confidence intervals (CI) of the log-10 of colony forming units (CFU)/mL, according to the different periodontal categories

	Periodontal health/gingivitis		Periodontitis stages I–II		Periodontitis stages III–IV		*p*-value
	Mean	95% CI	Mean	95% CI	Mean	95% CI	
*P. gingivalis*	2.97^bc^	(2.32–.54)	4.66^ac^	(4.03–5.30)	5.90^ab^	(5.20–6.48)	**<0.001**
*A. actinomycetemcomitans*	0.53^c^	(0.25–0.82)	0.70	(0.38–1.04)	1.17^a^	(0.72–1.64)	**0.044**
*E. nodatum*	1.18^bc^	(0.77–1.64)	2.34^ac^	(1.73–2.90)	4.15^ab^	(3.68–4.60)	**<0.001**
*T. forsythia*	4.97^c^	(3.97–6.11)	6.51^c^	(5.34–7.67)	9.24^ab^	(8.34–10.03)	**<0.001**
*T. denticola*	1.02^bc^	(0.64–1.40)	2.09^ac^	(1.64–2.53)	3.62^ab^	(3.15–4.23)	**<0.001**
*F. nucleatum*	5.73^c^	(4.97–6.43)	5.09^c^	(4.30–5.85)	7.07^ab^	(6.38–7.69)	**0.001**
*D. oralis*	1.74^c^	(1.04–2.44)	2.65^c^	(1.81–3.53)	5.16^ab^	(4.34–5.99)	**<0.001**
*F. alocis*	1.11^c^	(0.70–1.58)	1.61^c^	(1.12–2.09)	3.62^ab^	(3.10–4.09)	**<0.001**
*E. brachy*	4.44^c^	(3.61–5.25)	3.37^c^	(2.48–4.33)	6.24^ab^	(5.43–7.00)	**<0.001**
*E. saphenum*	2.61^bc^	(1.94–.3.32)	3.59^ac^	(2.85–4.32)	5.24^ab^	(4.49–5.93)	**<0.001**

All values are adjusted for the covariates that appear in each of the ANCOVA models (country, age, and current smoker). Adjustment for multiple comparisons using Sidak

^a^Statistically significant differences with the periodontal health/gingivitis group

^b^Statistically significant differences with the group stages I–II periodontitis

^c^Statistically significant differences with the group stages III–IV periodontitis

Statistically significant differences are marked in bold

In covariate analysis with the ANCOVA test, no assayed microorganism showed higher numbers in relation to gender and only *E. safenum* showed differences between smokers and no smokers (*p* < 0.05). For age, *F. nucleatum* presented differences in age (Supplementary Table S2). All culturable and unculturable microorganisms evidenced differences by country in ANCOVA analysis except *P. gingivalis* (*p* < 0.05) (Table S2).

**Fig. 3:**
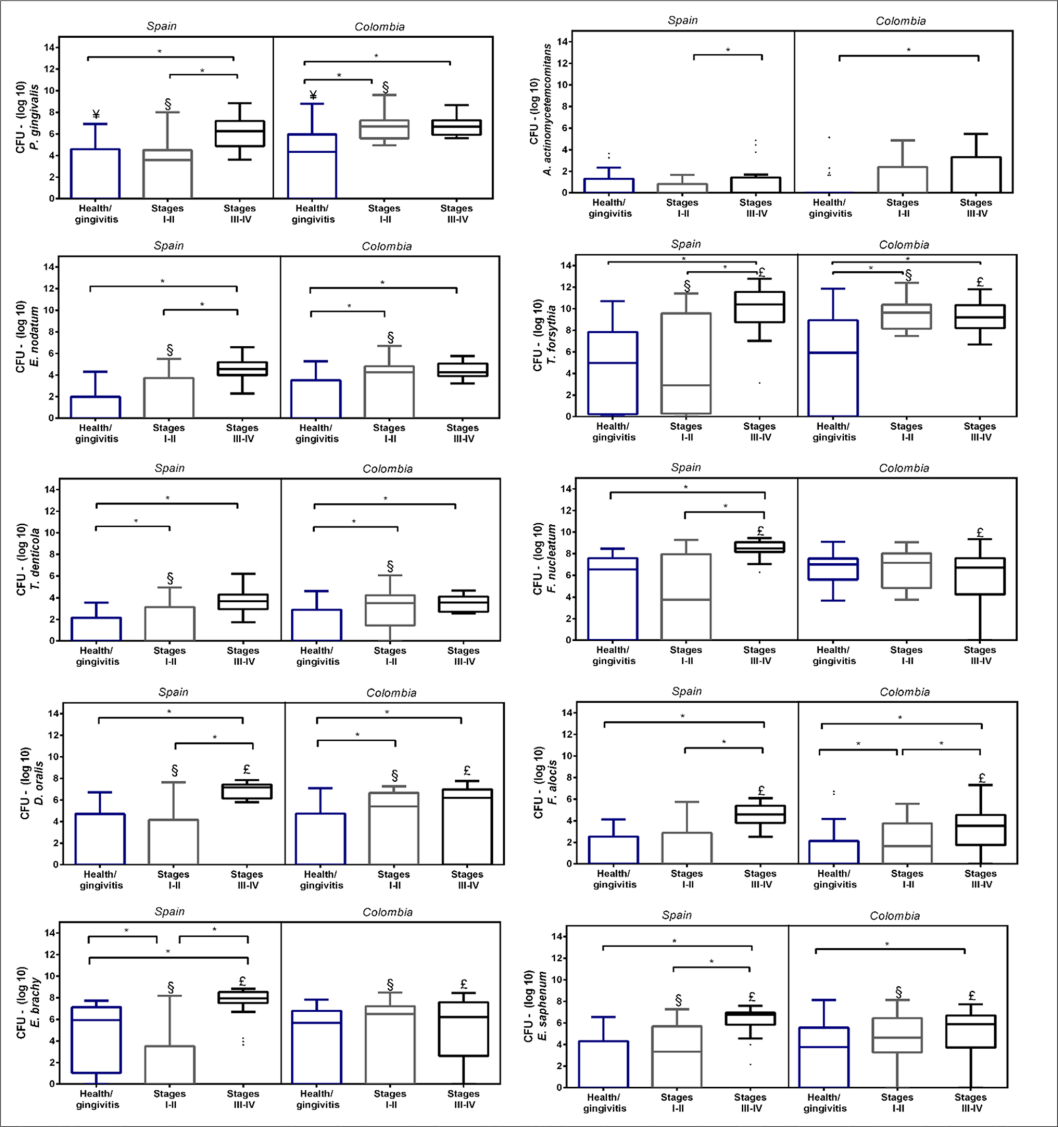
Box plot presenting concentrations, expressed as medians and interquartile ranges, of log-10 of colony forming units (CFU)/mL ranges of culturable and unculturable microorganisms. *Significant differences *p* < 0.05. ^¥^Significant differences *p* < 0.05 between countries—health/gingivitis subgroup; ^§^Significant differences *p* < 0.05 between countries—stages I–II subgroup; ^£^Significant differences *p* < 0.05 between countries—stages III–IV subgroup

Figure 3 depicts the quantitative analysis by stages and countries. All culturable and unculturable microorganisms showed higher concentration in health/gingivitis and/or stages I/II in Colombian samples except *A. actinomycetemcomitans*, *F. nucleatum*, and *F. alocis* (*p* < 0.05). In stages III/IV, the behavior was more homogeneous in culturable microorganisms without observing differences between the countries. However, in unculturable microorganisms, a higher concentration was observed in the Spanish samples (*p* < 0.05).

In both countries, *P. gingivalis*, *T. forsythia*, *D. oralis*, and *E. nodatum*, evidenced differences in the stages of periodontitis compared with health/gingivitis (*p* < 0.05). However, Spanish samples also evidenced differences among both stages of periodontitis (*p* < 0.05). *T. denticola* was similar between the countries with differences only among the health/gingivitis group vs. stages I/II (*p* < 0.05). *F. alocis* present a differential result among the countries; while in Colombia, the differences were significant between all groups; in the Spanish samples, no differences were observed between health/gingivitis and stages I/II groups. Interestingly, *E. brachy* and *F. nucleatum* only demonstrate differences in Spanish samples.

## Discussion

This microbiological observational study aimed to quantify the putative culturable and unculturable or difficult-to-cultivate target microorganisms with a sensitive qPCR assay comparing subgingival samples retrieved from subjects in Colombia and Spain categorized according to the new classification of periodontal diseases (2018) [1–3] using clinical and radiographic criteria. The obtained microbiological results clearly denoted a significantly higher number of these selected species, specifically, the target culturable bacteria *P. gingivalis*, *T. forsythia*, *T. denticola*, and *E. nodatum*, as well as the unculturable *F. alocis*, *E. saphenum*, *E. brachy*, and *D. oralis* in periodontitis samples in stages III/IV compared to health/gingivitis. In mild lesions, only cultivable microorganisms differentiated stages I/II of healthy/gingivitis. When comparing periodontitis samples according to their stage (stage I/II versus I/IV), target culturable bacteria were in significantly higher numbers in stage III/IV samples. However, the unculturable microorganisms except for *E. saphenum* did not show significant differences between stages. This association of the selected target culturable microorganisms (*P. gingivalis*, *T. forsythia*, *T. denticola*, and *E. nodatum*) with the severity of periodontitis has been corroborated by many clinical microbiological studies [5, 23, 24]. In fact, these association criteria have been one of the main criteria to identify the so-called periodontal pathogens [5]. In this study in two different populations, the prevalence of *A. actinomycetemcomitans* was very low. It, therefore, did not show an association with periodontitis. *A. actinomycetemcomitans*, a primary colonizer, associated with progressive lesions in periodontitis, is also associated with periodontitis. However, it does not differentiate between different phenotypes of periodontitis [25].

In a recent parallel pilot study, the microbiome of 60 subjects from the same sample pool used in the present study, with different periodontal conditions, from Spain and Colombia, was compared using next-generation sequencing (NGS) platforms (NGS) of 16 RNA genes. *F. alocis*, *Peptostreptococcaceae [Xi][G-4]* bacterium *HMT 369*, *Peptostreptococcaceae [XI][G-9] [Eubacterium] brachy*, *Peptostreptococcaceae [Xi][G-5] [Eubacterium] saphenum*, and *Desulfobulbus* sp. *HMT 041* were consistent microorganisms in stages III–IV periodontitis in both countries [26]. According to these results, these microorganisms were selected in this study. However, *Peptostreptococcaceae [Xi][G-4]* bacterium *HMT 369* could not be studied, because it corresponds to a phylum level whose species have not yet been established. The results of this qPCR-based study were consistent with the NGS-based study to detect culturable and difficult-to-cultivate bacteria [26]. Due to the high costs and technical complexity of NGS, using qPCR may allow the evaluation of large population samples, as was done in the present study.

It is a fact that NGS is a technique of choice for studying biofilm dysbiosis. This technique gives us relevant information about the diversity and composition of the microbiome. However, in a previous study, few microorganisms differentiate samples from healthy patients or those with gingivitis from periodontitis [26]. Microorganisms of the red complex were the microorganisms that increased during dysbiosis and were associated with the severity of periodontal lesions in the studied countries [26]. However, unculturable microorganisms such as *Filifactor alocis*, *Eubacterium saphenum*, *Eubacterium brachy*, and *Desulfobulbus oralis* in the dysbiotic biofilm have also shown essential differences for severe lesions [26].

Similarly, after several studies with NSG, significant coincidences have been observed in the increase of these non-culturable microorganisms in severe periodontitis in other populations [10–12]. However, these results must be validated in epidemiological studies with large population samples. NSG assesses the microbiome’s composition, but it is challenging to realize a multivariate analysis for epidemiological studies using bioinformatic analysis. Other techniques to validate its information, such as qPCR, have been used to quantify specific bacterial targets and used in cohort studies of intestinal microbiomes based on previous results of sequencing data [27, 28]. qPCR is the gold standard sensitive compared to NGS in fluid samples with low concentrations of bacteria [27]. This characteristic of qPCR could facilitate the study of microorganisms in the subgingival biofilm.

With the advent of molecular microbial diagnostics, previously unknown pathogens and pathobionts such as *D. oralis* or Gram-positive species of *Eubacterium*, belonging to the *Peptostreptococcus* family, as *E. nodatum*, *E. brachy*, and *E. safenum*, which have been associated with periodontitis [29, 30]. In the present study, these target unculturable bacteria (*F. alocis*, *E. saphenum*, *E. brachy*, and *D. oralis*) were also in higher concentrations in advanced periodontitis samples than in health/gingivitis. However, differences among stages were not established as in other culturable microorganisms.

Unculturable microorganisms were more consistent with advanced stages of periodontitis in the qualitative and quantitative analyses in this study. *F. alocis* has been suggested as a periodontal pathogen o due to these properties that enable this bacterium to colonize, survive, and outcompete other bacteria within the inflammatory environment of the periodontal pocket [30, 31]. Likewise, *D. oralis* is a novel human periodontal pathobiont that has been able to adapt to the subgingival environment. *D. oralis* are nonmotile Gram-negative bacilli, strict anaerobes, and difficult to cultivate. Proteomic and transcriptomic analysis revealed that most of the genes are actively expressed in the subgingival environment, and it can trigger a proinflammatory response in oral epithelial cells, suggesting a direct role in the development and progression of periodontitis [8].

*F. alocis* has been associated with periodontitis in India, Arabic, Mexico, and the USA populations using qPCR and conventional PCR [20, 32–34]. *F. alocis* lipoteichoic acid has recently been shown to induce the expression of proinflammatory cytokines (TNF-α, IL-6, IL-8, and MMP-2) in human gingival fibroblasts, similar to the lipopolysaccharide of historically associated Gram-negative bacteria with periodontitis, confirming the pathogenic potential in the development and progression of periodontitis associated with this bacterium [35] According to Cross et al., *D. oralis* has been isolated in coaggregation with *F. nucleatum* [8]. However, this could not be confirmed in this study.

The results are consistent with a similar association between these unculturable microorganisms with periodontitis using different microbial diagnostic methods in populations from North America [26, 36–38], Europe [10, 39, 40], Asia [11, 27, 41–43], and Latin American [44–46]. Other microorganisms associated with severe lesions were of the genus *Eubacterium* belonging to the *Peptostreptococcus* family, which *E. nodatum*, *E. brachy*, and *E. saphenum* have been isolated and characterized from periodontitis samples [47]. The etiological relevance of unculturable microorganisms in periodontitis and its impact on therapy has been recently investigated. Colombo et al. (2012) [48] reported the persistence of unculturable species such as *Bacteroidetes*, *Eubacterium* spp*.*, *F. alocis*, *P. micra*, and *Peptostreptococcus OT113* after mechanical periodontal therapy with adjunctive administration of amoxicillin and metronidazole. However, the relative importance of these species in the progression of periodontitis is still unknown.

When comparing the samples from Spain and Colombia, the Spanish population had significantly more smokers, while the Colombian population had more dental plaque and gingival bleeding. These differences did not significantly affect the concentration of microorganisms in the periodontitis group, although the samples from the Colombian healthy/gingivitis group harbored a higher concentration of culturable pathogens in healthy/gingivitis and stages I/II periodontitis compared with the equivalent Spanish samples. However, surprisingly, the Spanish samples showed a higher concentration of unculturable microorganisms. These differences can be explained by specific ecological pressures [44], and environmental factors such as food consumption or even socioeconomic factors or access to dental care [49, 50]. Another factor to consider is the greater diversity and richness of the subgingival microbiome in samples from Colombia, evidenced in our pilot study by NGS technic [26]. The concentration of microorganisms may be reduced by a more significant number of species found in the subgingival environment in the Colombian samples of stages III/IV. These ecologic changes among the geographic regions should be studied in the future.

## Conclusions

In spite of these limitations, the present investigation has associated the presence of a selected group of periodontal pathogenic bacteria, both culturable and unculturable, with periodontitis samples, more specifically with severe periodontitis, mainly the culturable species. More ecological studies are needed to disclose the pathogenic role of the unculturable microorganisms of the subgingival microbiome in the etiology, progression, and therapy response of periodontitis.

## Strengths and limitations

This study only evaluated nonculturable bacteria that showed similar associations among the countries evaluated in a previous pilot study performed with genome sequencing. Likewise, this report evaluates the classic culturable bacteria associated with periodontitis. Other unculturable microorganisms which could also be associated with periodontitis were not evaluated.

## Supplementary information

ESM 1: Supplementary Tables
