# Supplementary material for: Differential analysis of culturable and unculturable subgingival target microorganisms according to the stages of periodontitis
Source: Clin Oral Investig. 2023 Feb 18;27(6):3029–43. doi: 10.1007/s00784-023-04907-5 (PMC10264511; doi:10.1007/s00784-023-04907-5)
Supplement: Supplementary file 1 — Supplementary Tables [file 784_2023_4907_MOESM1_ESM.docx]

|  | **Independent variables** | **Unadjusted OR**  **(95% CI)** | **Adjusted OR**  **(95%** **CI)** | **LR (*p-*value)**  **BIC** |
| --- | --- | --- | --- | --- |
| ***P. gingivalis*** | **Country**  Spain  Colombia  **Groups**  Health/gingivitis  Periodontitis | 1.0 (Ref.)  **3.16 (1.51 - 6.59)**  1.0 (Ref.)  **3.69 (1.80 – 7.58)** | 1.0 (Ref.)  4.12 (1.38 – 12.21)  1.0 (Ref.)  4.75 (1.76 – 11.5) | LR (Chi^2^) = 0.98  Unadjusted BIC = 241.94  Adjusted BIC = 247.55  Difference = - 5.61 |
| ***T. forsythia*** | **Groups**  Health/gingivitis  Periodontitis | 1.0 (Ref.)  **3.0 (1.35 – 6.62)** | 1.0 (Ref.)  2.78 (0.94 – 8.19) | LR (Chi^2^) = 0.84  Unadjusted BIC = 173.19  Adjusted BIC = 178.34  Difference = - 5.15 |
| ***T. denticola*** | **Country**  Spain  Colombia  **Groups**  Health/gingivitis  Periodontitis | 1.0 (Ref.)  **2.33 (1.19 – 4.59)**  1.0 (Ref.)  **5.99 (2.99 – 12.0)** | 1.0 (Ref.)  1.55 (0.53 – 4.50)  1.0 (Ref.)  4.33 (1.68 – 11.1) | LR (Chi^2^) = 0.34  Unadjusted BIC = 221.93  Adjusted BIC = 226.22  Difference = - 4.29 |
| ***E. nodatum*** | **Groups**  Health/gingivitis  Periodontitis | 1.0 (Ref.)  **3.96 (2.01-7.78)** | 1.0 (Ref.)  2.68 (0.93 – 7.71) | LR (Chi^2^) = 0.39  Unadjusted BIC= 243.43  Adjusted BIC = 247.89  Difference = - 4.46 |
| ***D. oralis*** | **Groups**  Health/gingivitis  Periodontitis | 1.0 (Ref.)  **2.97 (1.53 – 5.74)** | 1.0 (Ref.)  2.59 (0.89 – 7.49) | LR (Chi^2^) = 0.75  Unadjusted BIC = 251.25  Adjusted BIC = 256.35  Difference = -5.1 |
| ***F. alocis*** | **Groups**  Health/gingivitis  Periodontitis | 1.0 (Ref.)  **3.89 (2.00 – 7.54)** | 1.0 (Ref.)  2.25 (0.91 – 5.56) | LR (Chi^2^) = 0.59  Unadjusted BIC = 228.42  Adjusted BIC = 233.32  Difference = - 15.07 |
| ***E. brachy*** | **Groups**  Health/gingivitis  Periodontitis | 1.0 (Ref.)  1.11 (0.58 – 2.16) | 1.0 (Ref.)  **1.31 (0.67 – 3.45)** | LR (Chi^2^) = 0.039  Unadjusted BIC = 227.58  Adjusted BIC = 224.44  Difference = 3.14 |
| ***E. saphenum*** | **Country**  Spain  Colombia  **Groups**  Health/gingivitis  Periodontitis | 1.0 (Ref.)  **2.42 (1.23 - 4.74**)  1.0 (Ref.)  **2.15 (1.09 – 4.24)** | 1.0 (Ref.)  1.94 (0.68 – 5.47)  1.0 (Ref.)  1.40 (0.53 – 3.69) | LR (Chi^2^) = 0.14  Unadjusted BIC = 231.43  Adjusted BIC = 237.99  Difference = - 6.56 |

**Table S1.** Multivariate analysis by non-conditional logistic regression for the detection of culturable and unculturable microorganisms by periodontal health and periodontitis status and country.

Model adjusted for country, age, and smoking.

OR = odds ratio; 95% CI = 95 % confidence interval; LR= likelihood ratio test; BIC= Bayesian information criterion. All models showed no differences in the likelihood ratio test except *E. brachy*. The unadjusted model should be reported for all models because they present a lower BIC^22^. *E. brachy* the adjusted model is reported. In bold the models accept. In bold are the significant results of the model selected.

**Table S2.** Multivariate analysis by ANCOVA for each microorganism evaluated.

|  | **F** | **p-value** | **F** | **p-value** |
| --- | --- | --- | --- | --- |
| **Dependent variable:** | ***P. gingivalis*** | | ***A. actinomycetemcomitans*** | |
| **Corrected model** | 13.011 | 0.000 | 1.792 | 0.117 |
| **country** | 18.246 | 0.000 | 1.799 | 0.182 |
| **Age** | 1.243 | 0.266 | 0.417 | 0.520 |
| **Smoke** | 2.377 | 0.125 | 0.104 | 0.748 |
| **Periodontal stage** | 19.091 | 0.000 | 3.174 | 0.044 |
| **Dependent variable:** | ***E. nodatum*** | | ***T. Forsythia*** | |
| **Corrected model** | 16.727 | 0.000 | 8.731 | 0.000 |
| **country** | 5.286 | 0.023 | 4.527 | 0.035 |
| **Age** | 0.707 | 0.402 | 0.416 | 0.520 |
| **Smoke** | 0.394 | 0.531 | 0 | 0.988 |
| **Periodontal stage** | 35.220 | 0.000 | 18.069 | 0.000 |
| **Dependent variable:** | ***T. denticola*** | | ***F. nucleatum*** | |
| **Corrected model** | 13.473 | 0.000 | 4.500 | 0.001 |
| **country** | 7.122 | 0.008 | 0.105 | 0.746 |
| **Age** | 0.337 | 0.562 | 8.308 | 0.004 |
| **Smoke** | 0.195 | 0.659 | 0.175 | 0.676 |
| **Periodontal stage** | 28.465 | 0.000 | 6.980 | 0.001 |
| **Dependent variable:** | ***D. oralis*** | | ***F. alocis*** | |
| **Corrected model** | 8.146 | 0.000 | 11.359 | 0.000 |
| **country** | 0.041 | 0.839 | 0.264 | 0.608 |
| **Age** | 1.159 | 0.283 | 0.876 | 0.351 |
| **Smoke** | 0.238 | 0.626 | 0.064 | 0.801 |
| **Periodontal stage** | 19.089 | 0.000 | 26.434 | 0.000 |
| **Dependent variable:** | ***E. brachy*** | | ***E. safenum*** | |
| **Corrected model** | 4.736 | 0.000 | 8.188 | 0.000 |
| **country** | 0.027 | 0.869 | 3.544 | 0.061 |
| **Age** | 0.224 | 0.637 | 1.955 | 0.164 |
| **Smoke** | 0.132 | 0.717 | 1.539 | 0.216 |
| **Periodontal stage** | 11.558 | 0.000 | 14.398 | 0.000 |

Adjusted for covariates country, age, smoke, periodontal stages
